# Query

**Published:** 1875-02

**Authors:** 


*Query.*

The senior editor will, if possible, answer in the next issue the question of Dr. J. C. H., viz.: What do you think of making doctors of women?

